# A high security double lock and key mechanism in HUH relaxases controls *oriT*-processing for plasmid conjugation

**DOI:** 10.1093/nar/gku741

**Published:** 2014-08-14

**Authors:** José Daniel Carballeira, Blanca González-Pérez, Gabriel Moncalián, Fernando de la Cruz

**Affiliations:** Departamento de Biología Molecular e Instituto de Biomedicina y Biotecnología de Cantabria, IBBTEC, Universidad de Cantabria-CSIC, C/Albert Einstein 22, 39011 Santander, Spain

## Abstract

Relaxases act as DNA selection sieves in conjugative plasmid transfer. Most plasmid relaxases belong to the HUH endonuclease family. TrwC, the relaxase of plasmid R388, is the prototype of the HUH relaxase family, which also includes TraI of plasmid F. In this article we demonstrate that TrwC processes its target *nic*-site by means of a highly secure double lock and key mechanism. It is controlled both by TrwC–DNA intermolecular interactions and by intramolecular DNA interactions between several *nic* nucleotides. The sequence specificity map of the interaction between TrwC and DNA was determined by systematic mutagenesis using degenerate oligonucleotide libraries. The specificity map reveals the minimal *nic* sequence requirements for R388-based conjugation. Some *nic*-site sequence variants were still able to form the U-turn shape at the *nic*-site necessary for TrwC processing, as observed by X-ray crystallography. Moreover, purified TrwC relaxase effectively cleaved ssDNA as well as dsDNA substrates containing these mutant sequences. Since TrwC is able to catalyze DNA integration in a *nic*-site-containing DNA molecule, characterization of *nic*-site functionally active sequence variants should improve the search quality of potential target sequences for relaxase-mediated integration in any target genome.

## INTRODUCTION

**Figure 1.**
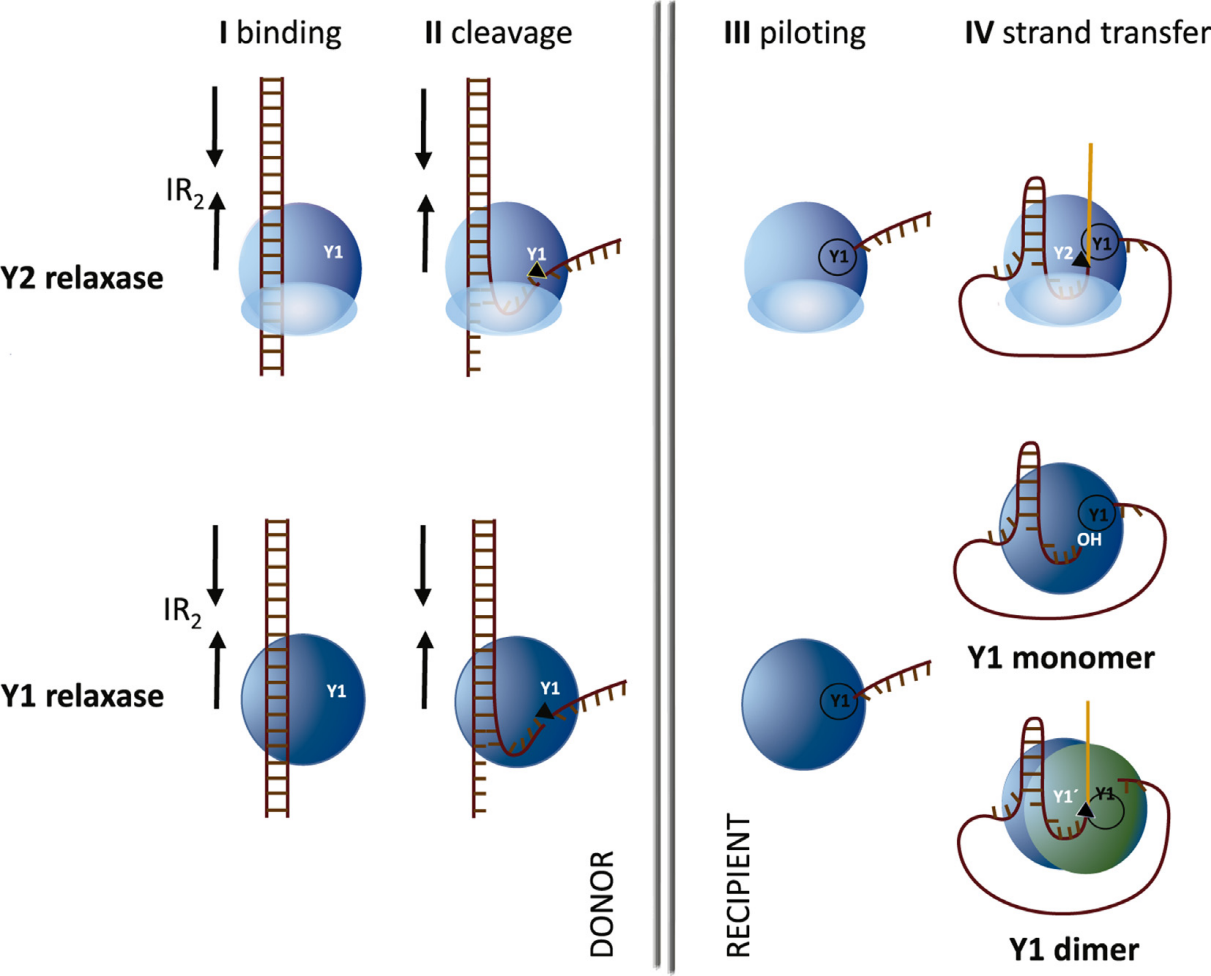
Models of conjugative DNA processing by Y1 or Y2 relaxases (modified from (7) and (8)). I, conjugation is initiated by the relaxase recognizing the proximal arm of an inverted repeat (IR_2_) adjacent to the *nic*-site. II, relaxase binding allows the formation of a single-stranded DNA (ssDNA) U-turn that positions the *nic-*site at the relaxase active site. The U-turn is stabilized by the ‘fingers’ subdomain of Y2 relaxases. After *nic*-cleavage, a covalent phosphotyrosine bond between the cleaved DNA strand and the relaxase is formed. III, subsequent DNA strand displacement generates the DNA single strand that, piloted by the relaxase, is transferred into the recipient cell. IV, In Y2 relaxases, a second tyrosine present in the same molecule attacks the newly formed *nic*-site to generate a free 3′OH end able to recircularize the transferred plasmid DNA. In Y1 relaxases, either the free 3′OH is released in the donor cell (monomeric Y1 model) or a second relaxase molecule provides the free tyrosine that attacks the newly formed *nic*-site (dimeric Y1 model).

Relaxases are DNA strand transferases responsible for the initial and final stages of DNA processing during bacterial conjugation, a mechanism of DNA transfer between bacterial cells (1). This process is biologically relevant due to its role in the diversification of bacterial genomes as well as in the dissemination of antibiotic resistance genes or virulence factors (2). Relaxases are part of the conjugative machinery and are encoded within conjugative plasmids. These plasmids, also called self-transmissible plasmids, of which more than 200 are reported in databases (3), generally present a mobilization (MOB) region which includes the origin of transfer (*oriT*) and the relaxase gene. Moreover, they contain a type IV secretion system (T4SS), which assures assembly and function of the mating channel. Other plasmids, called mobilizable plasmids, carry only the MOB region and need the presence of a helper conjugative plasmid to be transferred (4). The conjugative DNA transfer system can be divided into three functional modules (5): (i) a nucleoprotein complex called relaxosome, which consists of the *oriT* DNA, a relaxase and accessory nicking proteins, (ii) the transport channel, a multiprotein complex spanning the inner and outer membranes through which the substrate plasmid is secreted (6) and (iii) the conjugative coupling protein (T4CP), which is responsible for connecting the other two functional modules via protein–protein interactions and pumps the DNA into the recipient cell. The relaxase initiates conjugation by cleaving the *oriT* in a site- and strand-specific manner. The nicking reaction is mediated by a tyrosine residue that catalyzes a transesterification reaction (Figure 1). After cleavage, the relaxase remains covalently bound to the 5′ end of the cleaved DNA strand via a phosphotyrosyl linkage while the 3′ hydroxyl is sequestered by tight non-covalent interaction with the relaxase. The cleavage reaction is reversible. The tightly bound newly formed 3′ hydroxyl group can attack the 5′ phosphotyrosyl bond to regenerate the original molecule. However, when the relaxase-DNA complex releases the 3′-OH portion of the DNA (as after transport of the transferred strand into the recipient cell), a second tyrosine present in the same molecule or in a different molecule can attack a second *nic*-site (7,8), Figure 1). This last reaction takes place at the end of conjugation for regenerating the *oriT* in the recipient cell and is called strand-transfer reaction (9,10). A conjugation system is able to deliver large DNA molecules between donor and recipient cells, ranging from a few kb to several megabases. Relaxases control the conjugative process on the basis of their high specificity that permits binding to the correct site within the *oriT*, the so called *nic*-site, in presence of a huge excess of non-specific DNA. *nic*-site recognition is the key step of the conjugative process, i.e. the step in which relaxases selects the DNA that is to be transferred from cell to cell.

TrwC is the relaxase of the prototype plasmid R388. It is a relaxase of the MOB_F_ family, presently composed by 114 members and including relaxase TraI of plasmid F (4). TrwC presents two domains: relaxase and helicase. The relaxase domain is responsible for DNA recognition; it shows extremely high specificity towards its cognate *nic-*site. MOB_F_ relaxases belong to the HUH endonuclease superfamily (7). Catalytic residues include the HUH motif (His-hydrophobic-His) and a third histidine for metal coordination. Moreover, MOB_F_ relaxases have two catalytic tyrosines, involved in the initial cleavage reaction and the final intramolecular strand transfer reaction, respectively. Previous work, (8) showed that binding and cleavage are two distinguishable steps in conjugative DNA processing by TrwC. The two steps involve different sequence elements of the *nic-*site: the cleavage site and an adjacent inverted repeat (called IR_2_, Figure 1). The IR_2_ proximal arm and the nucleotides located between IR_2_ and the cleavage site are essential for supercoiled DNA (scDNA) processing. IR_2_ proximal arm recognition is crucial for the initial scDNA binding. Subsequent recognition of the adjacent ssDNA binding site was required to position the cleavage site in the active center of the protein so that the *nic*-cleavage reaction could take place. Similar observations were reported for the F plasmid relaxase TraI, which binds hairpin oligonucleotides in two distinct manners with different sequence specificities (11).

In addition to its role in conjugation, TrwC presents integrase activity in the recipient cell, being able to catalyze site-specific recombination between two different *nic*-site copies due to a site-specific recombination activity (12). Due to their property of catalysing DNA integration, relaxases and bacterial conjugation in general have been proposed as a tool for biotechnological applications (13,14).

In the present article we scanned R388 *nic-*site using full and partially randomized mutant libraries, depicting a sequence specificity map of the nucleoprotein interaction. Deeper analysis of the ssDNA region of the *nic*-site focused on the U-shaped turn located close to the catalytic site, which is conserved in the *nic*-site of the related plasmid F (15). We solved the X-ray structure of a TrwC nucleoprotein complex containing a *nic-*site mutant efficiently processed by TrwC that carries a different combination of nucleotides in the U-shaped turn. This structure underscores key features of the TrwC:DNA interaction. The TrwC *nic*-site specificity map allows knowledge-guided search of potential targets for HUH relaxases in any potentially useful target genome.

## MATERIALS AND METHODS

### Mutant library construction

Mutant libraries were constructed by an adaptation of the QuikChange™ method (16), using KOD Hot Start (Novagen) DNA polymerase and degenerate oligonucleotides (Sigma-Aldrich). Reactions were carried out in a total volume of 20 μl. Reaction mixtures were prepared according to the QuikChange™ manual, using 10 ng template plasmid DNA. Polymerase chain reaction (PCR) conditions were: initial denaturation for 3 min at 95°C (Hot Start) followed by 25 cycles of 1 min at 95°C, 1 min annealing in a gradient temperature between 49–55°C, 6 min extension at 68°C and 10 min final extension at 68°C. PCR products were then digested with *DpnI* (Fermentas) (10 units/2 h/37°C) and used to transform electrocompetent *Escherichia coli* DH5α (Nx^R^) cells carrying plasmid pSU711 (17). pSU711 is an *oriT*-deficient derivative of conjugative plasmid R388 (R388Δ*oriT*) that carries an otherwise complete conjugative machinery as well as a kanamycin resistance determinant (Km^R^) for selection.

#### Full randomization libraries of the nic-site

*nic-*site full randomization libraries were engineered in plasmid pSU4910 (Cm^R^), a vector carrying R388 *oriT*, using a modification of the QuikChange™ method. Degenerate primers were designed so that they carry N degeneracy (*N* = A,C,G,T) at desired positions. The *nic-*site was divided into eight randomization regions (A–H), each involving randomization (*N*) of up to five nucleotides. The number of randomized nucleotides was limited to a maximum of five in order to obtain exhaustive mutant libraries of affordable size and quality (18). Thus, libraries (A–H) include randomization of the nucleotide positions between brackets: A(1,2), B(3–7), C(8–11), D(12–16), E(17–20), F(21–25), G(26–28), H(29–31). Library design and numbering take the nucleoprotein complex resolved by X-ray diffraction (PDB ID: 1QX0; (19)) as reference.

#### Partially randomized libraries of the nic-site

Partially randomized libraries (R) were named by the first position number of the partially degenerate nucleotide that avoided the original nucleotide: R17 (17–21; HNNNN; H encodes for A,C,T), R23 (21–25; NNVNN; V encodes for A, C, G), R24 (22–26; NNDNN; D encodes for A, G, T) and (R20 19–22; NVNN) were designed in the quest for alternative sequences in the ssDNA region of the *nic-site*.

R-libraries were designed as fully randomized libraries but (i) considering codon degeneracies that do not encode all nucleotides in the desired positions and (ii) using as template a plasmid carrying a mutant *nic-*site not efficiently recognized by TrwC. The chosen plasmid was pSU4298, which contains a C24G *nic-*site mutation (20) leading to a conjugation frequency of 10^−5^. These codon degeneracies and template were used to avoid the presence of the wild-type (wt) sequence in the final library.

### Conjugation assays

**Figure 2.**
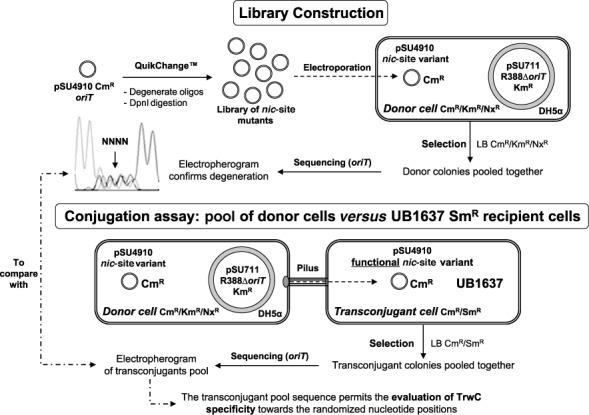
Experimental outline depicting the selection system for the study of TrwC specificity. Library construction: Quickchange allows the construction of a library of mutants at the *nic*-site. DH5*a* cells were transformed with the plasmid library by electroporation. Conjugation: mobilizable plasmids were selected by mating library-containing DH5a donor cells with recipient UB1637 cells. Pooled donor colonies as well as pooled transconjugant colonies were sequenced to be compared. See ‘Materials and Methods’ section for details.

Conjugation assays were performed as described (21) using UB1637 (Sm^R^) (22) as recipient cell and DH5α containing relevant plasmids as donor cells. Conjugation followed the same conditions as reported, using 1 h as conjugation time (20). After conjugation, transconjugants were pooled together, DNA extracted using a plasmid mini-prep kit (Sigma-Aldrich) and their *nic*-sites sequenced and compared with the electropherogram of the sequence of the pool of donors. A scheme of the experimental process is presented in Figure 2. In the case of the partially randomized libraries, isolated transconjugants were selected after conjugation and their DNA was extracted. *nic*-sites were sequenced and the different variants found were characterized in terms of conjugation frequency. DNA was extracted and used to transform new donors to obtain individual conjugation frequencies. Each experiment was repeated six times. Conjugation frequency was calculated as the number of transconjugants divided by the number of donor cells.

### Protein expression and purification

The relaxase domain of TrwC (TrwC_R_) was purified as described before (10,23–24). Briefly, pET23a: trwC-N293 was expressed in *E. coli* strain C43 (DE3) (25). Cells were lysed and the lysate centrifuged at 45 000 *g* for 45 min at 4°C. Protein purification was carried out in two chromatographic steps: P11-phosphocellulose (Whatman) and MonoS (Amersham), as described.

### Supercoiled DNA nicking assay

Reaction mixtures (20 μl) contained 200 ng of pSU4910 (containing wt *oriT*) or pSU4298IV (*nic-*site mutant IV) DNAs and different concentrations of TrwC (0, 0.01, 0.1 or 1 mg/ml) in buffer A (10 mM Tris-HCl pH 7.6, 50 mM NaCl, 0.02 mM ethylenediaminetetraacetic acid (EDTA) and 5 mM MgCl_2_). After incubation for 30 min at 37°C, reaction mixtures were digested with 1 mg/ml proteinase K (Roche) in 0.5% (w/v) sodium dodecylsulphate (SDS) for 15 min at 37°C. Reaction mixtures were applied to 0.8% (w/v) agarose gels containing 0.5 μg/ml ethidium bromide and electrophoresed at 100 V in 45 mM Tris-borate 0.5 mM EDTA buffer (pH 8.2). Bands were visualized in a BioRad Gel Doc system and quantified using Quantity One software.

### Oligonucleotide cleavage assay

Cleavage reaction mixtures contained 50 nM 5′-fluorescein-labeled oligonucleotides R(25 + 18) (5′GCGCACCGAAAGGTGCGTATTGTCT ATAGCCCAGATTTAAGGA3’) or RIV(25 + 18) (5′GCACCGAAAGGTGCGTATTCTTG GTAGCCCAGATTTAAGGA 3′) and variable concentrations of protein TrwC_R_ in 10 mM Tris-HCl, pH 7.6, 5 mM MgCl_2_, 110 mM NaCl and 20 mM EDTA. After incubation for 30 min at 37°C, digestion with 0.6 mg/ml proteinase K and 0.05% (w/v) SDS was carried out for 20 min at 37°C. Oligonucleotide separation and quantification were performed as described previously (10) using BioFocus^®^2000 capillary system (Bio-Rad).

### Complex formation, crystallization, X-ray data collection and processing

For the structural analysis of TrwC bound to mutant IV DNA, TrwC_R_ (at 6 mg/ml in 200 mM NaCl, 20 mM TrisHCl [pH 7.5], 1 mM dithiothreitol [DTT], 0.5 mM EDTA) and RIV(23 + 2) DNA substrate (5′ GCACCGAAAGGTGCGTATTCTTG GT-3′) were mixed at a 1:1.5 Protein:DNA molar ratio. Crystals (space group P6_5_) were grown with sitting-drop vapor diffusion at 22°C by mixing of 2 μl TrwC_R_–MIV complex with 1 μl reservoir solution A (2M ammonium phosphate, 0.1 M Tris-HCl [pH 8.5]). Data were collected at 105 K from a crystal transferred to cryoprotectant solution B (20% [v/v] ethylene glycol, 1.6 M ammonium phosphate, 80 mM Tris-HCl [pH 8.5]). Diffraction data to 2.4 Å was obtained at the European Synchrotron beamline BM16 and processed using MOSFLM and SCALA as part of the CCP4 package (26,27). Molecular replacement solutions in MolRep (CCP4) were obtained with the structure of TrwC_R_ bound to the 25-mer *nic*-site (19) as a search model (PDB ID: 1OMH). Refinement was performed in Phenix (28) and modeling in COOT (Crystallographic Object-Oriented Toolkit) (29).

## RESULTS

### Proximal IR_2_ and U sequences within the *nic*-region are essential for TrwC-mediated conjugative transfer

Resolution of cocrystal structures showed that the R388 *nic*-site includes a hairpin with a 4 nt loop and a 3′ ssDNA region which forms a U-shaped turn 5′ to the cleavage site. ((19), Figure 4). We have now scanned the *nic*-site by using full and partially randomized mutant libraries to discriminate the relative importance of each nucleotide for DNA conjugative processing. A scheme of the experiment is shown in Figure 2. Degenerate DNA libraries A to H were created to randomize the *nic-*site sequence as explained in ‘Materials and Methods’ section. These libraries were electroporated to *E.coli* DH5α competent cells until an appropriate number of transformants was obtained (18). Transformants were pooled and sequenced to determine whether the *nic*-site library was truly random. The pools were separately introduced in donor cells and subjected to plasmid conjugation assays. After 1 h mating with UB1637 recipients, mating mixtures were plated on LB agar plates containing the proper combination of antibiotics (Sm, Cm) to select for transconjugant colonies. Then cells were grown for 20 h at 37°C, pooled and sequenced. Comparison between the sequences of pooled transconjugant and pooled donor plasmid DNAs provides a representation of the nucleotide positions that have been selected (sieved) by TrwC activity (Supplementary Figure S1).

**Figure 3.**
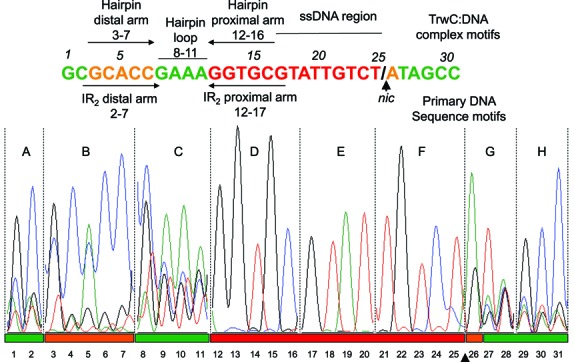
Full randomization libraries. Electropherogram showing the randomized region in the transconjugant pool sequence of each library tested (A–H). Essential nucleotide positions are colored in red, positions that admit only some mutations in orange and positions where any nucleotide can be located appear in green.

**Figure 4.**
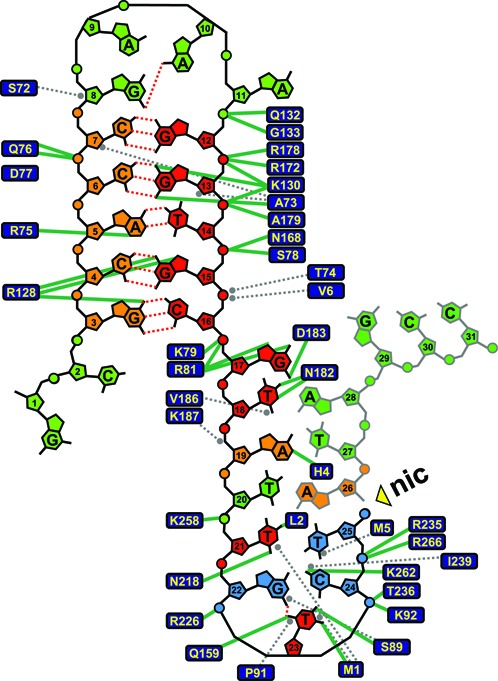
Classification of different specificity regions within the *nic* sequence according to the results obtained from randomization libraries. 2D representation of the nucleoprotein complex according to PDB ID: 1QX0 (19). Hydrogen bonds are indicated with green lines and van der Waals contacts with gray dashed lines. Nucleotides are colored like in Figure 3 according to the results obtained in the full randomization libraries plus those ones obtained from the partially randomized libraries of the ssDNA region. Nucleotides involved in the U-turn stabilization are colored in blue.

Figure 3 shows the electropherograms of each individual library pool (A–H). Only wt sequences were transferred in libraries D, E and F, while some mutations were allowed in libraries A, B, C, G and H. Analysis of the results allowed us to divide the *nic*-site sequence into separate regions (Figure 3). Some showed unique specificity for TrwC recognition, that is, only the wt sequence was detectable in the transconjugants (red colored nucleotides). Some others showed low specificity regions, that is, any sequence was transferred (green color). Finally, some others showed an intermediate situation, in which some sequence modifications but not others were accepted by TrwC (orange colored nucleotides).

According to the results shown in Figure 3, nucleotides 12–25 (red), namely the proximal arm of IR_2_ (nucleotides 12–16) and the ssDNA region before *nic* (nucleotides 17–25), appear to be essential for TrwC processing *in vivo*, as only the wt sequence was transferred to recipient cells. Conversely, some mutations in nucleotides 3–7, which form part of IR_2_ distal arm, were efficiently transferred.

Nucleotide position 26, just 3′ to the nick position, has to be a purine (A or G) to be efficiently transferred *in vivo*. Furthermore, a *nic*-site containing any nucleotide at positions 1–2, 8–11 (hairpin loop) or 27–32 was efficiently processed by TrwC. These results fit well with the degree of interaction of each nucleotide with TrwC in the X-ray structure of the nucleoprotein complex (19). The allowed substitutions are also in good agreement with the proposed DNA processing model where TrwC recognizes only the proximal arm of dsDNA plasmid in the donor cell, while the distal arm is required to a minor extent for IR_2_ hairpin formation in the recipient cell ((8), Figure 1).

However, some mutants in the essential sequence 12–25, and particularly in the ssDNA region (19-27), could also be efficiently transferred, although with a lower conjugation frequency than the wt *nic*. These sequences may be masked in the assay due to overrepresentation of the wt *nic* sequence in the library. In the construction of the libraries, the wt *oriT* is used as template for QuikChange™ and some wt background is always present (18). In order to solve this problem, partially randomized mutant libraries that ensure the absence of the wt *nic* sequence in the assay were designed.

### Partially randomized libraries: beyond the original ssDNA (19-27) *nic*-site sequence

To avoid the presence of wt sequences, a new set of mutant libraries was obtained by the QuikChange method using primers carrying DNA degeneracies (B, D, H, V) that encode three out of four possible nucleotides at the desired position, while excluding the wt nucleotide. Besides, they contain any nucleotide (N) around the restricted position to create diversity. Moreover, a plasmid carrying a mutant *nic-*site not efficiently conjugated by TrwC (pSU4298 C24G *nic*-site, conjugation frequency 10^−5^) was used as template for the PCR reaction, in order to prevent the presence of the wt plasmid as a result of under-digestion of hemi-methylated species by *DpnI* (30).

**Figure 5.**
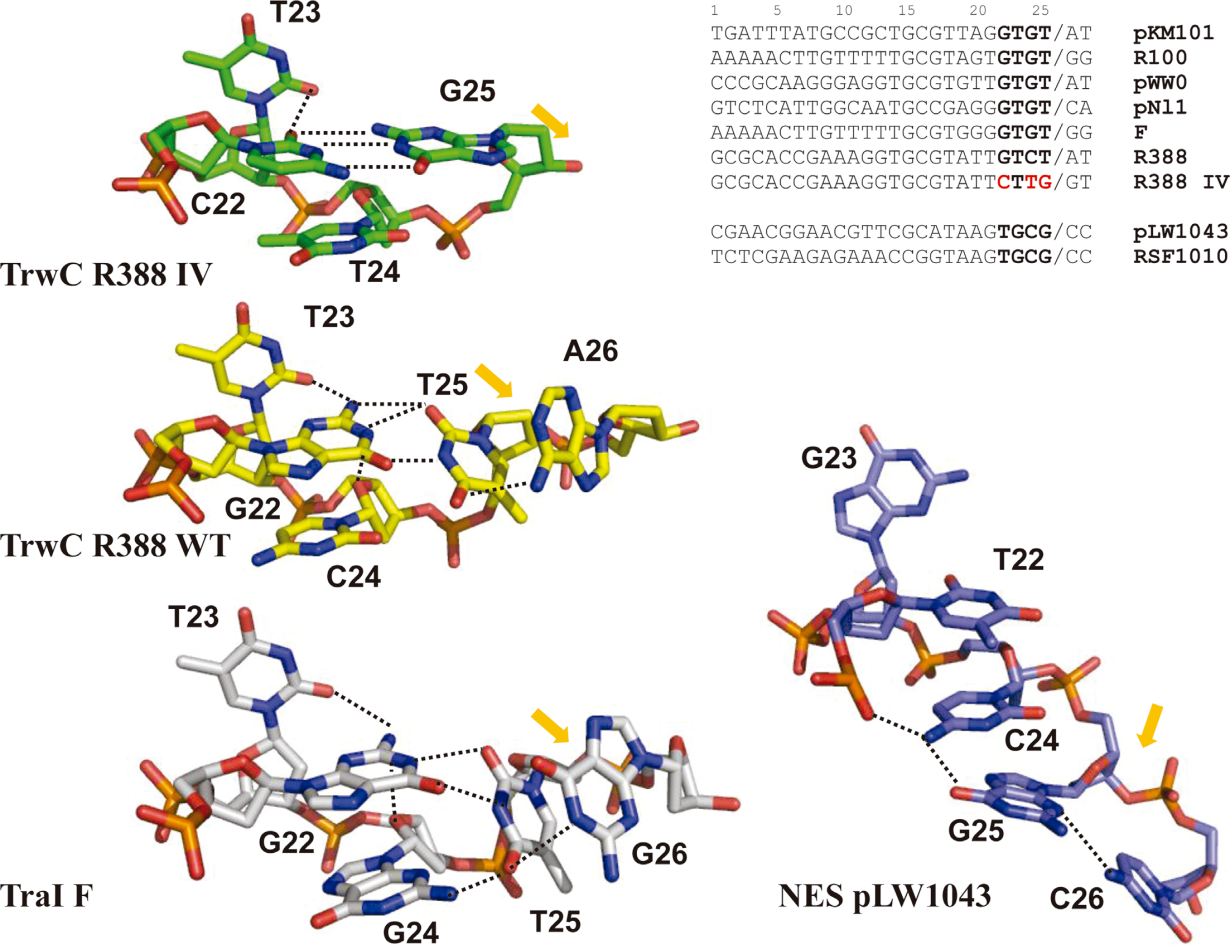
Representation of the U-shaped turns in the structures of the nucleoprotein complexes R388_TrwC_R_:mutant IV, R388_TrwC_R_:*nic*-site (PDB ID: 2CDM,(23));F_TraI:*nic*-site (PDB ID: 2A0I (31)) and pLW1043_NES: *nic*-site (PDB ID: 4HT4 (32). The nucleotides are numbered equivalent to R388_TrwC structure. The position of the *nic*-cleavage site is highlighted by an orange arrow. Hydrogen bonds are represented by dashed lines. The alignment of representative *nic*-sites of MOB_F_ and MOB_Q_ plasmids is shown. U-shaped turn forming nucleotides are shown in bold while nucleotides changed in mutant IV are shown in red.

Although a guanine is located in an equivalent position than C24 in R388 in some MOB_F_ plasmids (Figure 5), TrwC cannot efficiently transfer G24-*nic* containing plasmids. Lys262 in TrwC was shown to discriminate between specific nucleotides in position 24 (20). To better characterize the nucleotides allowed at position 24, a library was constructed that randomized five nucleotides (24-28) using NNDNN degeneration, where D in position 24 allows the presence of any nucleotide excluding the original C (D: A, G or T). This partially randomized library with a fixed nucleotide at position 24 will be called library R24 (restricted in 24). We will use this nomenclature pattern for all partially randomized libraries constructed in this work.

Theoretical library R24 contains 768 out of the 1024 sequences involved in full randomization of the five positions and no wt *nic-*site. Around 7 000 colonies were obtained after transformation. This library size was considered representative of the theoretical diversity (18). These colonies were pooled together and conjugated to UB1637 recipients. Thirty transconjugant colonies were selected and sequenced. Once the different sequences obtained were characterized, the individual conjugation frequencies (transconjugants per donor) were calculated. Interestingly, only five different mutant sequences were found, as shown in Table 1.

**Table 1.**
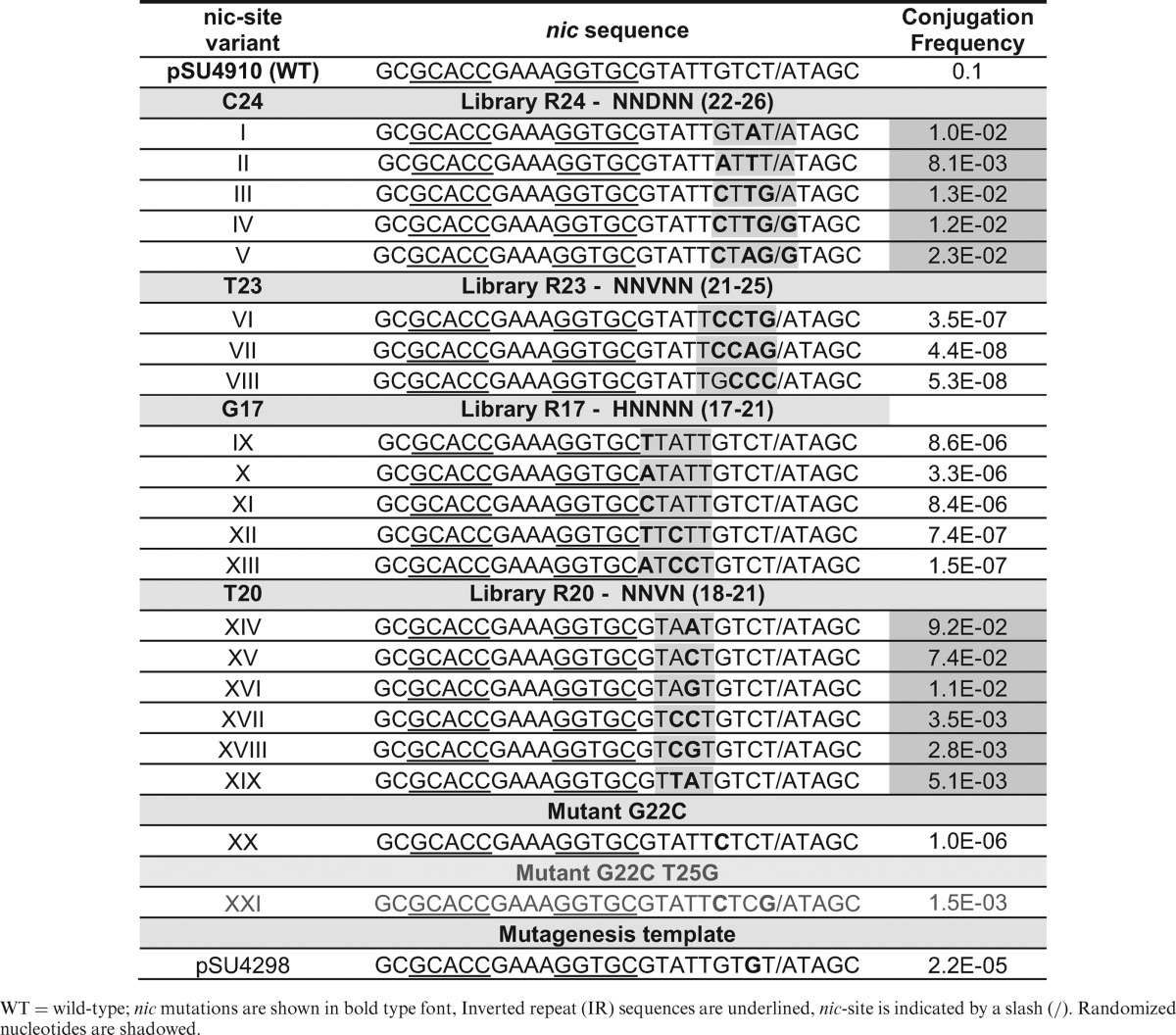
Conjugation frequencies of the characterized *nic* sequence mutants found in partially randomized libraries designed to avoid the wt sequence

WT = wild-type; *nic* mutations are shown in bold type font, Inverted repeat (IR) sequences are underlined, *nic-*site is indicated by a slash (/). Randomized nucleotides are shadowed.

The selected mutant sequences (I–V) displayed conjugation frequencies 10-fold below the wt sequence. It can therefore be assumed that mutants carrying up to four mutations are efficiently processed by TrwC. According to these results, C24 can be mutated to Adenine with a 10-fold decrease in the conjugation frequency (mutant I). It can also be mutated to Thymine when other surrounding nucleotides are also mutated (mutants II, III and IV). The conjugation frequency of mutant C24A was only 5-fold lower than wt when some other nucleotides were also mutated (mutant V). However, the C24G transversion was not observed in any of the analyzed transconjugants. All transconjugant mutants found in this library have a purine base at position 26, as previously observed in the full randomization assays. Interestingly, most MOB_F_ conjugative plasmids also present a purine in the equivalent position (Figure 5).

None of the R24 mutants display a mutation in position 23. To determine whether this position is really critical, a new library, similar to library R24 but centered on nucleotide T23 (library R23) was constructed. In this case, the randomization was NNVNN, where V encodes A, C or G, but not T, forcing position T23 to be changed during PCR. After repeating the same analysis as for library R24, only three different transconjugant sequences (VI–VIII) were found (Table 1). In all sequences VI–VIII, the same T23C mutation was present, together with some accompanying mutations. Nucleotide T21 remains invariant in all mutants from library R23. Despite being detectable by conjugation of the R23 library, mutants VI, VII and VIII displayed extremely low conjugation frequencies, 10^6^-fold lower than wt (Table 1), highlighting the role of T23 for proper TrwC-mediated DNA transfer. Unexpectedly, mutations G22C and T25G found in sequences III, IV and V from library R24 were also present in variants VI and VII, indicating that this combination may in some way support functional interaction with TrwC. To determine whether transversion G22C could be effectively processed by TrwC, we mobilized the corresponding mutant DNA (Mutant XX, Table 1). However, the mobilization frequency was low (10^−6^ transconjugants per donor), highlighting the role of the second mutation T25G for proper transfer by compensation of G22C mutation.

To analyze T24 contribution in U-turn formation by C22 and G25, we obtained a plasmid containing a new *oriT* mutant (XXI) with G22C and T25G mutations, but the native C24 nucleotide. This mutant was transferred at a conjugation frequency only 10× lower than mutant IV, but 1000× higher than mutant G22C (Table 1). This result shows that just T25C mutation is able to compensate G22C mutation.

T23 presents three hydrogen bond interactions with TrwC, between specific atoms in the nitrogenous base of the nucleotide and residues Met1, Ser89 and Gln159, as well as van der Waals interactions with Met1 and Pro91 (19). The only other nucleotide in the *nic* region that presents a triple hydrogen bond interaction with TrwC in the nucleoprotein complex is G17, which makes an H-bond with Asp183 and a double H-bond with Arg81. We therefore assayed the effects of varying position G17 using a new partially randomized library: R17 (nucleotides 17–21), carrying the randomization HNNNN to avoid hairpin modification (nucleotides 12–16), where H could be A, C or T. Poor conjugation frequencies, of around 10^−7^, were observed in the five mutant sequences (IX–XIII) that were able to be transferred in library R17 (Table 1). Among the five mutants of library R17, T18 as well as T21 remained unchanged. Remarkably, in the X-ray structure of the TrwC–DNA complex, the nitrogenous base of T21 is confined to a reduced pocket delineated by the side chains of Met1, Leu2 (H-bond), Arg190, Asp216 and Asn218(H-bond). A van der Waals interaction with Met1side-chain blocks any potential interaction of nucleotide 21 with nucleotides 22–25. T21 interactions with T20 are also highly improbable. A similar situation can be described for T18, with two hydrogen bond interactions with Asn182 and a van der Waals contact with Val186.

G17, T18, T21 and T23 show strong interactions with TrwC and cannot be mutated without a decrease in the conjugation frequency. No other mutation in the *nic* sequence could compensate for mutation of these critical residues. Moreover, G17, T18 and T23 are conserved in most *nic* sequences of the MOB_F_ family, while T21 change to G21 only occur in pKM101 and F plasmids (Figure 5).

Conversely to G17, T18, T21 and T23, nucleotide T20 presents no direct interaction with TrwC. Thus, in the partially randomized library R20 (NNVN in positions 18–22) any nucleotide at position 20 could be efficiently processed by TrwC (Table 1).

In summary, partially randomized libraries provided valuable additional information for the characterization of the *nic* sequence. According to the information retrieved from the full and partial randomization assays, a refined two dimensional representation of the TrwC:DNA-specific interactions in the nucleoprotein complex could be inferred (Figure 4). Two main motifs are needed for efficient DNA processing by TrwC, namely the hairpin formed by IR_2_ upon binding with TrwC, which is important for stabilization of the complex, and the U-shaped motif needed for proper accommodation of the *nic-*site for cleavage. In Figure 4, nucleotides that are red colored are critical, while any mutation is allowed at green-colored sites. Orange nucleotide positions represent an intermediate situation, in which some mutations are allowed while others are highly detrimental. Nucleotides involved in the U-turn stabilization are colored in blue.

### The *nic*-site in mutant IV can be positioned at the active site of TrwC

*Nic-site* mutants III, IV and V, are still efficiently transferred although they present 3–4 mutations around the cleavage site, affecting the nucleotides involved in the U-shaped structure. These mutants are particularly interesting for the analysis of the mechanism by which TrwC recognizes the *nic-*site. Therefore, we solved the X-ray structure of the complex of TrwC with mutant IV *nic*-site (Supplementary Table S1, PDB ID: 4PCB).

As nucleotides 1 and 2 were neither interacting with TrwC relaxase domain (TrwC_R_) nor forming the hairpin structure, RIV(23 + 2) instead of RIV25 + 2 oligonucleotides were used. Moreover, although all previous TrwC structures were obtained with selenomethionine derivatives of TrwC_R_, we were able to obtain native crystals with TrwC_R_-mutant IV. Although the structure of the complex was obtained with precipitant conditions different than those originally used, crystal packing (space group P6_5_) was the same as in the former structures 1OSB and 2CDM (19,23). Thus the overall structure of the TrwC_R_-IV complex was almost identical to those reported (root mean square deviation for the Ca atoms is 0.62 Å between TrwC_R_-IV structure and 2CDM), except it contained phosphate ions instead of sulphate ions. The main difference revealed by the new structure was the formation of a different U-shaped structure, in which the formation of three hydrogen bonds between G22C and T25G in TrwC_R_-IV (Figure 5 and Supplementary Figure S2) stabilize the U-turn shape DNA structure. G22, C24 and T25 orientation in the complex is mainly stabilized by base stacking and by H-bonds with T23. The only specific interactions with TrwC are G22 with Ser89 and Lys262 and C24 with Lys262 (Supplementary Figure S3A). In the IV mutant, both Lys262 and Ser89 interact now with G25 and Lys262 with T24 (Supplementary Figure S3B). These protein–DNA interactions together with maintenance of the T23 interactions with the protein and the Watson–Crick base pairing between C22 and G25 allows the formation of the U-shape structure in TrwC_R_-IV.

The U-shaped structures of relaxase:DNA complexes crystallized to date are shown in Figure 5. In all MOB_F_ structures, T23 is extruded from the extended ssDNA by its tight binding to the relaxase. This conformational change allows the distortion of the ssDNA chain to generate the U-turn. In TraI_F:*nic-*site and TrwC_R388 wt, nucleotides 22 and 24, locate their nitrogenous base rings in parallel planes, forming an apparent π–π stacking interaction. ssDNA bending, driven by the stacking of two non-contiguous nucleotides, is also conserved in the structure of the single-tyrosine MOB_Q_ relaxase NES where G26 (G23 using the same numbering as in R388) is out of the ssDNA base packing, thus allowing the stacking of T22 and C24 (pLW1043 in Figure 5). This stacking bends the DNA to orient the *nic-*cleavage site in NES although, in the crystal structure,G25 does not interact with T22 and the U-turn is not completely formed. It was observed that C24 and G25 stacked with the corresponding G25 and C24 in another molecule in the crystal (33). In summary, all HUH relaxases share a ssDNA bending mechanism where nucleotide 23 is swung out of the ssDNA. The interaction 22:25 makes the U-turn more pronounced in the crystal structures of MOB_F_ relaxases with DNA.

### Mutant IV-containing ssDNA or scDNA are efficiently cleaved by TrwC_R_*in vitro*

**Figure 6.**
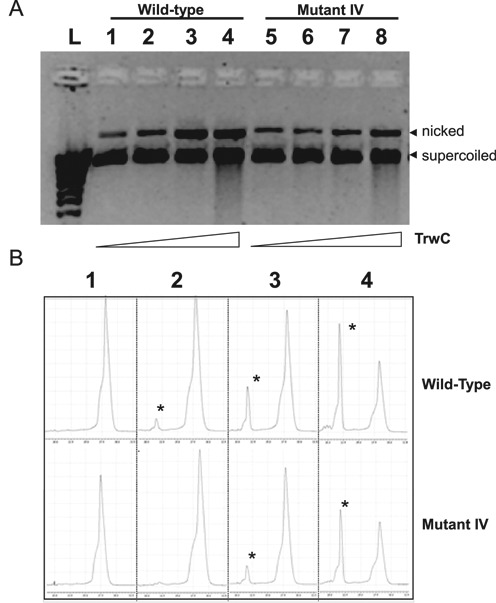
(**A**) Supercoiled DNA cleavage of wild-type *nic* sequence and *nic* mutant IV by TrwC. Reactions were carried out as described in ‘Material and Methods’ section and products resolved in a 0.8% agarose gel. Growing concentrations of purified TrwC_R_ were added to reaction mixtures containing pSU4910 (wild-type) or mutant IV SC DNA (200 ng): lane L, DNA ladder; lanes 1 and 5, no TrwC_R_ added, lanes 2 and 6, TrwC_R_ 0.3 μM; lanes 3 and 7, TrwC_R_ 3 μM; lanes 4 and 8, TrwC_R_ 30 μM;. The position of nicked DNA and SC DNA is indicated. (**B**) Single stranded oligonucleotide cleavage assays followed by capillary electrophoresis. Growing concentrations of purified TrwC_R_ were added to reaction mixtures containing 50 nm 5′-fluorescein-labeled oligonucleotides R(25 + 18) (wild-type) or RIV(25 + 18) (Mutant IV). (1) no TrwC_R_ added; (2) TrwC_R_ 0.3 μM; (3) TrwC_R_ 3 μM; (4) TrwC_R_ 30 μM. The positions of the cleaved oligonucleotides are shown by asterisks.

To correlate *in vivo* mobilization of mutant IV with TrwC processing activity, we analyzed *in vitro* TrwC cleavage activity using mutant IV-containing ssDNA or scDNA substrates. In both cases, *nic*-cleavage was carried out by incubating each substrate with increasing concentrations of the relaxase domain of TrwC (TrwC_R_, residues 1–293) and digesting the protein that remains covalently attached to the substrate to release the cleavage products. *In vitro* assays of scDNA cleavage mimic the TrwC DNA processing reaction on plasmid DNA in the donor cell. Relaxation of scDNA was analyzed as described in ‘Materials and Methods’ section using purified TrwC_R_ and the same pSU4910 or pSU4298IV plasmids as those used for mobilization (Table 1). As observed in Figure 6A, the mutant IV containing scDNA is slightly less prone to *in vitro* relaxation by TrwC than the scDNA containing the wt *nic*-site, although TrwC can still relax mutant IV scDNA.

ssDNA oligonucleotides R(25 + 18) and RIV(25 + 18) (‘Materials and Methods’ section) were used to analyze DNA binding and cleavage by TrwC. These ssDNA oligonucleotides mimic the ssDNA that is transferred into the recipient cell. TrwC_R_ is known to cleave *in vitro* oligonucleotides containing the *nic*-site, resulting in two products that can be analyzed by capillary electrophoresis (8). As shown in Figure 6B, TrwC is able to cleave a mutant IV-containing oligonucleotide RIV(25 + 18). However, the yield of cleaved products is lower than using the wt *nic*-site oligonucleotide R(25 + 18). 61% of wt and 55% of mutant IV oligonucleotides are cleaved by TrwC_R_ 30 μM after incubation for 30 min at 37°C. The cleavage assays in comparison with the wt *nic-site* sequence performed *in vitro* lead again to the same conclusions than the conjugation experiments *in vivo*.

## DISCUSSION

Relaxases need to act as DNA sieves during conjugation to avoid undesired transfer of non-cognate DNA sequences, including spurious sequences in the bacterial chromosome. They achieve this goal by exquisitely controlling recognition of the *nic*-site. Most relaxases belong to the so-called HUH protein superfamily (7), with the only remarkable exception of the BamHI-like relaxases of the MOB_C_ family (34). In this article, we analyzed TrwC, the relaxase of plasmid R388 and probably the best known prototype of the HUH relaxase protein family. We propose that the interaction of TrwC with its cognate *nic*-site occurs by a highly specific lock (TrwC) and key (*nic*) interaction. The *nic* sequence contains two distinct motifs needed to habilitate DNA processing by TrwC (8): the inverted repeat sequence IR_2_ and a single stranded DNA sequence encompassing the cleavage site. *nic*-sequence comparisons suggest that similar motifs exist in other HUH relaxases, underlying a common recognition mechanism (7).

In this work, we used saturating libraries of variant *nic* sequences to extract the sequence information used by TrwC in *nic*-site recognition. Full randomization library screening showed that nucleotides of the distal arm of IR_2_ can accept some changes (Figure 3) without lowering the conjugation efficiency. Although formation of a stable hairpin seems important for the process, not all five nucleotides of the distal arm have to be complementary with those of the proximal arm. Similar results were observed by (8) using site-directed mutants. In fact, an inverted repeat is not found at the *nic*-site sequence of some other plasmids containing HUH relaxases (19,35). The four nucleotides of the loop within IR_2_ (GAAA, nt 8–11) can be exchanged for any other nucleotide without affecting conjugation. This result is supported by the crystal structure of the TrwC-*nic*-site complex where the GAAA loop does not show any interaction with TrwC. Conversely, the proximal arm of the hairpin is crucial for TrwC recognition as only the wt sequence was found after full randomization. These nucleotides display strong interactions with TrwC amino acid residues, the double hydrogen bond interactions with the nitrogenous bases in G13 and G15 being especially remarkable. These results are in good agreement with the proposed DNA processing model (Figure 1) where TrwC recognizes only the proximal arm of dsDNA plasmid in the donor cell, being the distal arm required to a minor extent for IR_2_ hairpin formation in the recipient cell (8).

Nucleotides following IR_2_ and up to the *nic*-site (nucleotides 17–25), were sieved with high precision by TrwC, according to the full randomization assays (Figure 3). The extended ssDNA region 17–21 is almost invariant due to the high number of specific interactions with the protein. Only position 20 can accept all possible nucleotides (Table 1). However, assays using partially randomized libraries over nt 22–25 unveiled that other sequences besides the wt can be efficiently used by TrwC (Table 1). Nucleotides 22–25 are responsible for the formation of the U-shape structure that serves to accommodate the phosphodiester bond to be cleaved in the proper position of the active site. The sequence 22–25 has a conserved configuration (GTGT) in most MOB_F_ plasmids (Figure 5), while displaying transversion GTCT in R388. It was also suggested by point and double mutations that a three-base interaction (similar to the interaction between G22, C24 and T25 in R388) might be required to position the scissile phosphate for cleavage in F plasmid (15). Although nucleotide G22 is critical, as inferred from the fact that its change abolishes conjugation (mutant XX, Table 1), double mutants G22C and T25G could be mobilized (mutants III–V, Table 1). In the three dimensional (3D) structure of the TrwCR–MIV complex, there is an obvious new interaction, a triple hydrogen bond between C22 and G25 that makes the structure resemble in size and charge distribution of the U-shaped turn of the wt complex (Figure 5). In the wt relaxase–DNA complexes of F (31) and R388 (19), the only MOB_F_ relaxases resolved to date with bound DNA, nucleotides 22–25 are located in a similar position (Figure 5).

When the structure of MOB_F_ complexes was compared with the MOB_Q_ relaxase–DNA complex (32), the absence of the sharp 180° U-shape turn in the MOB_Q_ complex is apparent (Figure 5). MOB_F_ relaxases, also called Y2 relaxases, contain two catalytic tyrosines, one involved in the initial cleavage reaction and the other in the terminal reaction. In fact, two paths were described for the DNA onto the TrwC molecule. Although two pairs of conserved tyrosines have been found in MOB_F_ relaxases, the position of the catalytic tyrosines is not conserved in the three MOB_F_ structures solved, despite their high sequence and structural similarity (19,36–37). The functional differences are also remarked by the absence of sequence-specific DNA binding detected for the pCU1 relaxase (37).

In Y2 relaxases the U-turn region of the *nic*-site is held by a helical subdomain, called fingers by analogy with DNA polymerases (19). Thus, the fingers domain as well as the sharp U-turn by 22:25 interaction seems to be necessary in Y2 relaxases to allow the intramolecular DNA strand transfer reaction.

On the other hand, in MOB_Q_ relaxases the fingers subdomain is not found and only one catalytic Tyr was described (Y1). Due to the presence of only one active tyrosine in MOB_Q_ and other Y1 relaxases, two alternative versions of the recircularization mode have been proposed ((7) and Figure 1): (i) a free 3′OH end released in the donor cell is able to attack the phosphotyrosine bond and recircularize the plasmid (monomeric Y1 model) and (ii) a different molecule with a free tyrosine attacks a second nic-site and generates the free 3′OH end for the subsequent recircularization reaction (dimeric Y1 model). Thus, in the dimeric model, the *nic*-site has to be accessible to two Tyr coming from adjacent molecules. The ssDNA configuration found in the pLW1043 NES-DNA structure suggests that, due to the absence of the fingers subdomain, the *nic*-site is not invariably oriented toward the catalytic site of the relaxase and stacks with another molecule in the crystal (33). Although there is no evidence that NES forms a dimer, its 3D structure suggests a dimeric Y1 model where the ssDNA U-turn could be located between two subunits of a dimeric relaxase in a way that the *nic*-site could be cleaved by the catalytic tyr of the adjacent subunit.

In summary, the refined interaction between the *nic*-site, a huge, flexible DNA substrate, and the HUH relaxases, represents an excellent mechanistic example of enzyme specificity, where intra and inter-molecular interactions play synergistic contributing roles. Despite this highly secure mechanism, alternative functional sequences were found by partially randomized mutagenesis. This finding broadens the number of potential targets for HUH relaxases, thus increasing the number of integration sites in a given target genome.

Although new genome editing technologies, such as zinc finger nucleases, transcription activator-like effectors and the CRISPR/Cas9 system (38) have been recently developed, the only systems for site-specific DNA integration in human cells that have been used as tools in gene therapy are provided by viruses. Virus-mediated integration of genes at undesired cryptic sites is still a major problem (39). One could speculate that, by design, nature-selected viral integration sites are simpler than conjugative *oriTs*. There is no harm (from the virus point of view) of an occasional error in the integration site. This might be lethal for the host, but is irrelevant for the virus. In the conjugative case, acting on an erroneous sequence leads to transfer of the wrong sequence, and thus affects the fitness of the plasmid. *oriT* sequences have been selected by evolution as exquisite selective systems of DNA recognition and processing. TrwC relaxase recognizes a DNA sequence of about 20 nucleotides and shows high specificity toward it, resulting a priori in a good candidate for biotechnological applications. Moreover, it was recently proposed that several human genome sequences are potential TrwC targets (40). Results from this work indicate that the non-uniform 3D shape of DNA (41) may result in most targets being either non-recognizable or non-accessible to relaxase processing, thus reducing the number of potentially recognizable sequences to those where the *nic*-sequence conformation resembles its 3D structural shape when located in the structural context of the *oriT*. This difference makes relaxase enzymes attractive for genomic engineering. Our study provides for the first time a comprehensive analysis of the nucleotides required for intermolecular interactions with the relaxase and intramolecular interactions with other nucleotides. Once the specificity map of wt TrwC is defined, protein engineering of TrwC or any other relaxase could improve recognition and processing of alternative DNA sequences present in potential target genomes.

## SUPPLEMENTARY DATA

Supplementary Data are available at NAR Online.

SUPPLEMENTARY DATA

## ACCESSION NUMBER

PDB accession code: 4PCB.
