# Supplementary material for: A high security double lock and key mechanism in HUH relaxases controls oriT-processing for plasmid conjugation
Source: Nucleic Acids Res. 2014 Aug 14;42(16):10632–43. doi: 10.1093/nar/gku741 (PMC4176350; doi:10.1093/nar/gku741)
Supplement: SUPPLEMENTARY DATA [file supp_42_16_10632__index.html]

A high security double lock and key mechanism in HUH relaxases controls oriT-processing for plasmid conjugation — A high security double lock and key mechanism in HUH relaxases controls oriT-processing for plasmid conjugation — SUPPLEMENTARY DATA 

# A high security double lock and key mechanism in HUH relaxases controls *oriT*-processing for plasmid conjugation

## SUPPLEMENTARY DATA

**Files in this Data Supplement:**

- SUPPLEMENTARY DATA
