# Supplementary material for: A high security double lock and key mechanism in HUH relaxases controls oriT-processing for plasmid conjugation
Source: Nucleic Acids Res. 2014 Aug 14;42(16):10632–43. doi: 10.1093/nar/gku741 (PMC4176350; doi:10.1093/nar/gku741)
Supplement: SUPPLEMENTARY DATA [file supp_gku741_nar-01011-z-2014-File008.pdf]

Table S1. Crystallographic data collection and analysis

Space group	$P6_5$
Unit cell dimensions	
a	148.7
b	148.7
c	77.8
Data range (last shell)	66-2.5 (2.57-2.50)
Observations (unique)	340996 (36116)
Completeness (%) (last shell)	99.8 (99.6)
R^{a}_{sym} (last shell)	0.124 (0.418)
Non-hydrogen atoms (solvent molecules)	5356 (109)
R_{cryst}^{b} (last shell)	0.255 (0.295)
R_{free}^{c} (last shell)	0.294 (0.336)
r.m.s. bond length (A˚)	0.011
r.m.s. bond angles (deg.)	1.447

[a] Rsym is the unweighted R value on I between symmetry mates.
[b] Rcryst=$\Sigma_{hkl}((F_{obs}(hkl))-(F_{calc}(hkl))/\Sigma_{hkl}(F_{obs}(hkl))$.
[c] Rcryst is the cross-validation R factor for 5% of reflections against which the model was not refined.

FIGURE S1

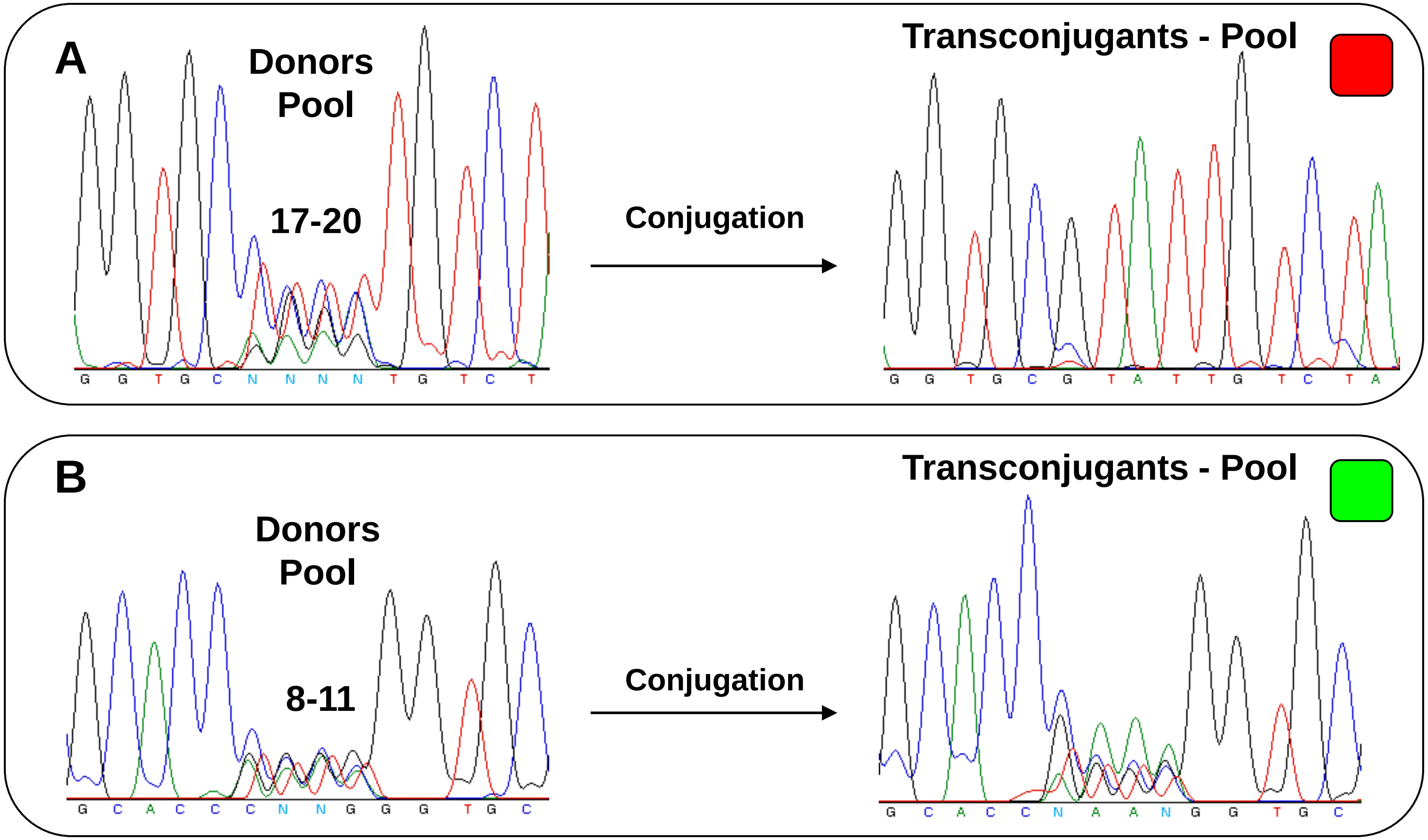
A
Donors
Pool
17-20
Conjugation
Transconjugants - Pool
G G T G C N N N N T G T C T
G G T G C G T A T T G T C T A
B
Donors
Pool
8-11
Conjugation
Transconjugants - Pool
G C A C C C N N G G G T G C
G C A C C N A A N G G T G C

FIGURE S2

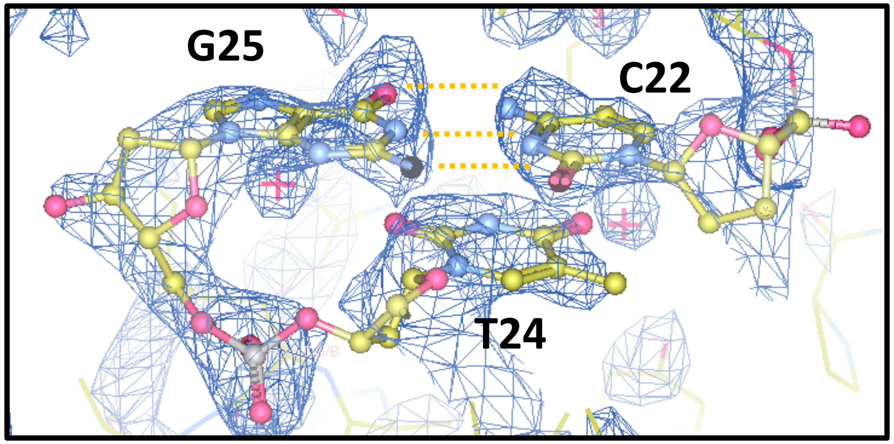

FIGURE S3

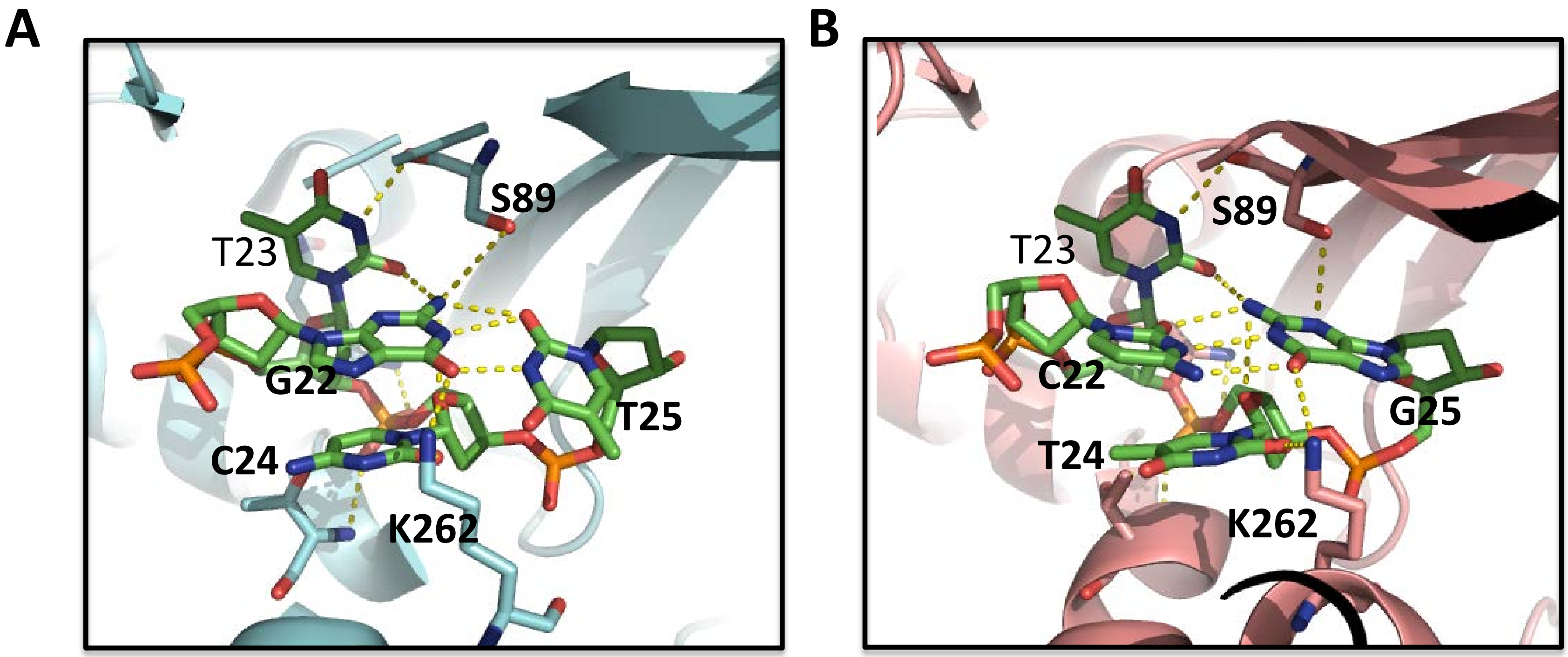

SUPPLEMENTARY FIGURE LEGENDS

Figure S1. Comparison between the pool of donors from a mutagenesis library of the *nic* sequence and the pool of transconjugants obtained from its mating assay. The differences between them depend on TrwC activity. The profile of the transconjugant pool permits the classification of the nucleotides randomized, if both donor and transconjugant sequences present a similar shape the region is not filtered, i.e. it is not important for TrwC processing (green colour). On the other hand, if the conjugation assay of a degenerated library gives as transconjugant pool only the wild-type, that means that the given region is important for TrwC activity (red coloured). The nucleotides that present an intermediate situation between both are coloured in orange.

Figure S2. X-ray structure of the complex between TrwC wild-type and mutant IV. The 2.5 Å resolution 2Fo-Fc electron density map contoured at 1 σ shows the U-turn region of the $TrwC_R$ bound DNA. The three hydrogen bonds between C22 and G25 are shown.

Figure S3. Details of TrwC-DNA intermolecular interactions and DNA-DNA intramolecular interactions responsible of U-turn formation in TrwC-WT (A) or TrwC-IV (B) complexes. TrwC protein is depicted as cyan (A) or salmon (B) cartoons. Interacting amino acids and nucleotides are shown as sticks. Yellow dotted lines indicate hydrogen bonds.
